# Host-pathogen interactions in periodontitis: an integrative interkingdom perspective

**DOI:** 10.3389/fimmu.2026.1797726

**Published:** 2026-04-02

**Authors:** Lina Janeth Suarez, Paula K. Vargas-Sanchez, Nikola Angelov, Eleftherios Mylonakis, Roger M. Arce

**Affiliations:** 1 Departamento de Ciencias Básicas y Medicina Oral, Universidad Nacional de Colombia, Bogotá, Colombia; 2 Centro de Investigaciones Odontológicas, Pontificia Universidad Javeriana, Bogotá, Colombia; 3 Clinical Epidemiology Unit/UNIECLO, School of Dentistry, Universidad El Bosque, Bogotá, Colombia; 4 UT Houston Department of Periodontics and Oral Hygiene, University of Texas School of Dentistry, Houston, TX, United States; 5 Department of Medicine, Houston Methodist Research Institute, Houston, TX, United States

**Keywords:** biofilms, dysbiosis, host-pathogen interactions, mouth microbiota, periodontitis

## Abstract

Periodontitis is an infectious, inflammatory, non-communicable disease characterized by tissue destruction driven by host responses to dysbiotic shifts in oral microbial communities. The subgingival microbiome constitutes a complex ecosystem in which bacteria, fungi, viruses, and archaea interact via interkingdom communication to modulate the inflammatory response through molecular mechanisms that remain largely unknown. This narrative review aims to understand how functional imbalances within the microbiome alter the microenvironment and promote uncontrolled inflammation responsible for periodontal tissue damage, with implications for systemic disease. The search strategy was conducted according to the PRISM-S extension, to include studies evaluating interkingdom host-pathogen interactions at the gingiva interphase leading to microbial and immune dysbiosis. The discovery of fungi acting as opportunistic pathogens highlights their role in enhancing biofilm virulence and exacerbating host responses, contributing to the total inflammatory burden. Similarly, viruses and archaea influence bacterial metabolism through mechanisms including lysis, nutrient recycling, horizontal gene transfer, and interspecies hydrogen transfer. This interkingdom crosstalk disrupts symbiosis, facilitating enhanced biofilm formation, increased production of virulence factors, and antibiotic resistance. A better understanding of the interkingdom perspective necessitates a comprehensive polymicrobial approach to diagnosis and treatment that extends beyond simply controlling bacteria to include the modulation of interkingdom communication systems. Developing new therapeutic alternatives that address these complex interactions is essential for improving outcomes achieved with mechanical therapy and managing the interrelationships between periodontitis and other systemic diseases.

## 1 Introduction

Periodontitis is a highly prevalent chronic inflammatory condition worldwide, affecting the tooth-supporting structures and leading to progressive tissue destruction and eventual tooth loss (1). The accumulation of microbial plaque on tooth surfaces primarily initiates the disease, triggering a complex host inflammatory response (2, 3). Historically, the understanding of periodontitis etiology focused predominantly on specific bacterial pathogens. Early investigations in oral microbiology identified various anaerobic bacteria in supragingival and subgingival plaque, establishing a growing list of putative periodontal pathogens as key to unlocking the disease’s microbial origins. Influential studies (4, 5) shaped thinking around periodontal microbiology for many years, establishing the concept that complexes of specific subgingival bacteria, notably the “red complex” (comprising *Porphyromonas gingivalis*, *Treponema denticola*, and *Tannerella forsythia*), directly correlated with clinical periodontal status.

However, advances in periodontal microbiology have moved beyond a single-pathogen or “red complex” paradigm toward a more comprehensive polymicrobial synergy and dysbiosis (PSD) model. The more contemporary PSD model posits that a synergistic microbial community, not individual pathogens in isolation, causes periodontitis by disrupting host-microbe homeostasis (6). Contemporary science now views the etiology of periodontitis more as a dysbiotic change—characterized by an alteration in the abundance or influence of individual species within the polymicrobial community—rather than a classic infection of single microbial etiology (7–10). The chronic nature of interkingdom dysbiosis can subvert or exhaust the host immune response, leading to a pathological cycle where immune mediators drive tissue destruction (11).

The host response in periodontitis is fundamentally characterized by an uncontrolled inflammatory response to the persistent microbial colonization of tooth surfaces and gingival tissues (12). The inflammatory cascade, triggered by the dysbiotic shifts in oral microbial communities, is the primary driver of tissue destruction (13). The host functions not as a passive recipient of microbial attack but as an active participant, in which a dysregulated or excessive immune response significantly contributes not only to the progression of periodontitis but also promotes local and systemic dysbiotic changes (14, 15). The mediators (IL1, TNFα, IL-17, IL-6, IL-23, RANKL, OPG, MMPs, PGE2, etc.) regulate inflammatory signals that control local inflammation and tissue destruction, actively stimulating bone resorption (16, 17).

A defining characteristic of periodontitis is the establishment of a positive feedback loop between oral microbial dysbiosis and the host inflammatory response (18). The dynamic relationship effectively bypasses the question of whether dysbiosis initiates inflammation or vice versa, and instead emphasizes reciprocal reinforcement as the actual driver of periodontitis. Within the pathological cycle, the dysbiotic community functions as a quasi-organismal entity that communicates via sophisticated physical and chemical signals to enable polymicrobial synergy (19, 20). Consequently, the inflammatory response becomes ineffective, uncontrolled, and destructive in the susceptible host (11). Dysregulated inflammation inadvertently creates an environment characterized by altered redox potential, increased nutrient availability from tissue breakdown, and changes in local pH (20). The environmental alterations further favor the proliferation and virulence of pathogenic species, thereby deepening the dysbiotic state. The self-perpetuating cycle ensures that periodontitis remains a chronic condition in which interkingdom dysbiosis and host inflammation continuously exacerbate one another (21).

Investigating the emerging etiological paradigms of periodontitis—specifically the polymicrobial interkingdom challenge, the self-perpetuating positive feedback loop between dysbiosis and inflammation, and the genetic determinants mediating host susceptibility to infection and exacerbated inflammatory responses—remains essential for catalyzing a shift in clinical management (3). Comprehending such multifaceted mechanisms facilitates the development of advanced diagnostic and therapeutic strategies designed to extend beyond merely controlling specific bacterial pathogens (22). Instead, the more contemporary approach should aim to restore ecological balance within the oral microbiome and the host, mandating the adoption of a broader, integrated strategy to significantly improve treatment outcomes (23). To comprehensively explore these concepts, this manuscript was structured as a narrative review following the PRISMA-S extension to report transparency in the literature search strategy. Full details regarding the databases searched, search strings utilized, and the completed PRISMA-S checklist are available as a **Supplement file**.

## 2 The emerging interkingdom perspective in periodontitis pathogenesis

The microbiome is defined as the ecological communities of microorganisms—including bacteria, archaea, fungi, viruses, and parasites—that cohabit specific environments of the human body. This definition encompasses the microbiota (the microorganisms themselves), their collective genome (including plasmids and transposons), their metabolic products, and the physical conditions of the inhabited microenvironment (24). Because bacteria are numerically the most prominent microorganisms in the human body, the term “microbiome” is often used synonymously with the “bacterial microbiome,” although these entities are inherently distinct and defined by their complex interrelationships and host interactions (25). While bacterial contributions remain central to periodontitis pathogenesis, the oral microbiome is recognized as an intricate multikingdom ecosystem encompassing diverse microorganisms, including bacteria, fungi, viruses, and archaea. Together with the human host, this integrated network functions as a “holobiont”—a complex, co-evolving ecological unit wherein host tissues and multikingdom microbial communities collectively determine states of health or disease. A comprehensive understanding of periodontitis now acknowledges the significant relevance of these non-bacterial microorganisms in potentially driving dysbiotic changes within the subgingival microenvironment (26).

**Figure 1:**
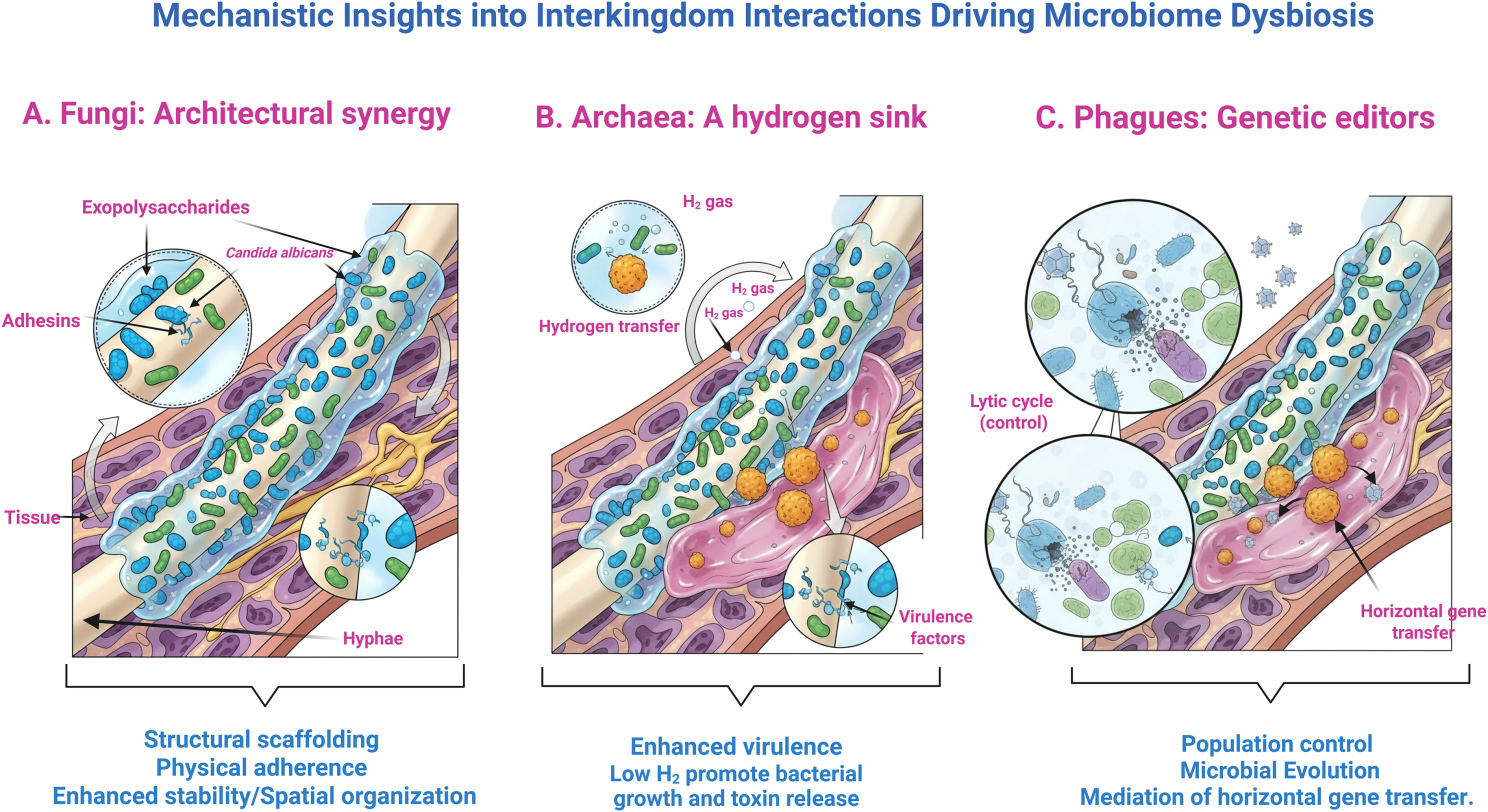
Synergistic interactions between bacterial and non-bacterial microorganisms disrupt host homeostasis through three primary axes. **(A)** Candida albicans hyphae and exopolysaccharides establish physical scaffolds that facilitate bacterial adherence and enhance biofilm stability. **(B)** Methanogenic archaea function as hydrogen sinks; the removal of metabolic H2 stimulates bacterial proliferation, toxin release, and the expression of virulence factors. **(C)** Bacteriophages regulate the ecosystem through lytic-cycle-mediated population control and facilitate horizontal gene transfer, mechanisms that accelerate microbial evolution and the dissemination of resistance or virulence traits. Collectively, these diverse kingdoms converge to transition a healthy microbiome into a dysbiotic state characterized by increased tissue adherence, enhanced toxicity, and the accelerated evolution of pathobionts.

The majority of common, well-studied bacteria, particularly those relevant to the human microbiome and ecological systems, fall into four major phyla: Bacillota (formerly Firmicutes), Pseudomonadota (formerly Proteobacteria), Bacteroidota (formerly Bacteroidetes), and Actinomycetota (formerly Actinobacteria). Other important bacterial phyla in humans include Chlamydiota (Chlamydiae) and Spirochaetota (Spirochaetes), as well as the Candidate Phyla Radiation (CPR), an expansive, superphylum within the domain Bacteria (27). The CPR group accounts for a significant portion (up to 25%) of all bacterial diversity (28). Similarly, Phyla exist for fungi and archaea, while the classification of viruses is still evolving. **Figure 1** summarize the periodontal “non-bacterial” Microbiome, its interactions with the bacteriome, and how they contribute to dysbiosis.

### 2.1 From composition to dysbiosis: mapping the subgingival microbiome in periodontitis

The oral cavity hosts one of the most diverse microbiomes in the human body, second only to the gastrointestinal tract (29). Beyond the 700 bacterial species present, this community represents a highly diverse phylogenetic landscape encompassing various non-bacterial domains and entities (30). Within this ecosystem, the subgingival microenvironment presents unique ecological challenges. Anaerobic bacteria, including key genera such as *Porphyromonas*, *Prevotella*, and *Fusobacterium*, predominantly colonize this low-oxygen space (31). The low oxygen tension characteristic of the subgingival crevice acts as a critical factor that actively selects for particular microbial communities. It favors the growth of anaerobes and influences the metabolic activities of other microbial kingdoms, which in turn benefit from metabolic cross-feeding (32–34). These dynamics indicate that the microenvironment functions not as a passive site of infection, but as an active selective pressure that shapes the composition and intricate interkingdom interactions of the resident microbiome (35).

**Figure 2:**
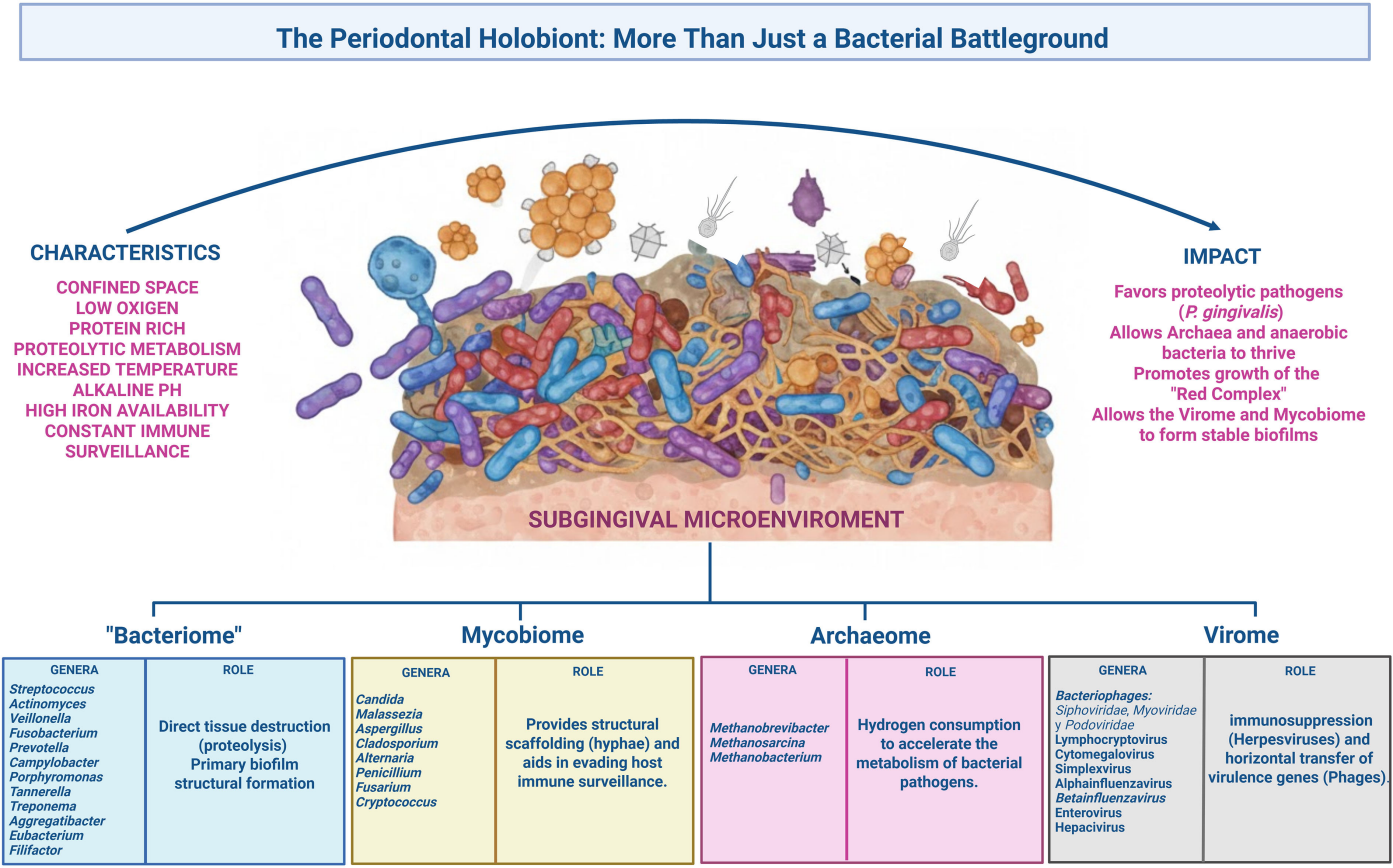
The periodontal holobiont constitutes a complex multikingdom ecosystem wherein specific subgingival environmental characteristics, including low oxygen tension and protein-rich substrates, facilitate the transition toward a pathogenic state. Within this niche, the bacteriome initiates direct tissue destruction through proteolytic metabolism, while the mycobiome, particularly *Candida* species, establishes essential structural scaffolding via hyphae to facilitate bacterial adhesion and evade host immune surveillance. Simultaneously, the archaeome functions as a metabolic catalyst; methanogens such as *Methanobrevibacter* consume hydrogen to promote the proliferation of fermenting bacterial pathogens. Furthermore, the virome contributes to pathogenesis through herpesvirus-mediated immunosuppression and the bacteriophage-mediated horizontal transfer of virulence genes. Collectively, these interkingdom interactions favor the expansion of Red Complex proteolytic pathogens, such as *Porphyromonas gingivalis*, thereby promoting the development of a stable, highly virulent biofilm.

Oral microbial dysbiosis (a harmful shift in the relative abundances and individual components of the microbiome) acts as a significant and well-established factor in the pathogenesis of oral diseases, including periodontitis (13, 36, 37). Studies reveal distinct subgingival microbial community structures in periodontitis that differentiate diseased states from healthy conditions (7, 38). An increased burden of pathogenic or pathosymbiont microbes within the oral ecosystem fundamentally characterizes this shift towards dysbiosis (39). This environment functions as the periodontal holobiont—a complex multikingdom ecosystem wherein specific subgingival environmental characteristics (including low oxygen tension and protein-rich substrates) drive the transition toward a pathogenic state (**Figure 2**).

The fungal component of the oral microbiota, known as the oral mycobiome, represents a numerically minor component compared with bacteria but remains functionally significant within mucosal and biofilm ecosystems. Culture-independent surveys consistently identify *Candida* spp. as the dominant fungal taxa, with *Candida albicans* usually representing the most prevalent species. Other genera, such as *Malassezia*, *Saccharomyces*, and *Cladosporium*, occur at lower abundances and exhibit niche specificity, including within the subgingival sulcus (40, 41). Structural and chemical synergy characterizes the interaction between the mycobiome and the bacteriome, enhancing the pathogenicity of the subgingival biofilm. Fungi, particularly *Candida* species (*albicans* and non-*albicans*), serve as architectural scaffolds (42). Their hyphal networks provide a physical surface for the attachment of non-motile bacteria (43–46), facilitating the maturation of multi-kingdom biofilms. The mycobiome modulates bacterial behavior; *Candida* hyphae, for instance, enable the adhesion of bacteria that otherwise lack efficient attachment mechanisms. This interaction creates a community with significantly heightened resistance to both antibiotics and host immune clearance (47–49). For example, distinct interactions occur between *Escherichia coli* and the fungus *Candida tropicalis* in extraoral conditions, such as Crohn’s disease. These interactions lead to thicker biofilms that induce a high-magnitude inflammatory response and a level of pathogenicity unattainable by the bacterial component alone (50, 51).

Beyond physical support, fungi engage in metabolic cross-talk with bacteria. For example, bacterial signaling molecules regulate the yeast-to-hyphae transition. Specifically, exogenous quorum-sensing molecules (QSMs) from *Pseudomonas aeruginosa*—such as 3-oxo-C12-homoserine lactone and various phenazines like pyocyanin (52)—suppress the transition of *Candida albicans* into its hyphal form, while fungal metabolites provide carbon sources for bacterial growth (53, 54). This partnership also promotes immune evasion, as fungal extracellular matrices sequester bacteria, shielding them from host neutrophils and antimicrobial agents (55, 56). Consequently, the host’s genetic susceptibility to fungal colonization creates a permissive environment for these synergistic interactions, ultimately exacerbating the inflammatory destruction of the periodontium (57, 58).

The oral cavity also hosts a diverse virome, particularly human herpesviruses such as Herpes Simplex Virus Type 1 (HSV-1), Epstein-Barr virus (EBV), and Cytomegalovirus (CMV), which contribute to oral pathologies and gingival inflammation (59, 60). These viruses function not merely as passive residents; specific viral agents engage in complex synergistic interactions with bacterial periopathogens, profoundly influencing the initiation and progression of periodontitis (31, 61). The interaction between the bacteriome and the virome (specifically bacteriophages) represents a sophisticated ecological synergy (61). To understand their role in disease initiation, one must examine the early dysbiotic phase. In periodontally healthy sites, the phageome consists mostly of lytic bacteriophages that regulate the bacterial community structure and inhibit pathogenic overgrowth (62). However, during the early transition to dysbiosis, lysogenic phage communities become increasingly common. By integrating into bacterial genomes, these phages facilitate horizontal gene transfer (63), disseminating virulence factors (64) and antibiotic resistance genes throughout the subgingival biofilm (65). This introduction of novel genetic material into previously benign bacteria contributes to the transition of commensals into pathogenic phenotypes, making phages compelling therapeutic targets (66–68). Through density-dependent regulation, phages create niche space that allows rare and potentially pathogenic organisms to flourish, thereby sustaining the ecosystem in a state of constant ecological imbalance (69, 70). Furthermore, the extensive bacterial lysis resulting from viral activity releases immunogenic cell wall fragments, such as lipopolysaccharides and bacterial DNA, which stimulate host receptors and exacerbate the inflammatory response (66, 71, 72).

Within this complex landscape, the dual role of phages in the oral ecosystem (acting as both commensals and pathogens) indicates their significant influence on the ecology of the human oral microbiome (73); the phageome functions as a natural regulator to keep bacterial populations in check (62, 74, 75). The virome, therefore, contributes to dysbiosis by modulating microbial community structure through selective predation and the introduction of novel functional traits (76, 77). This viral-mediated genetic plasticity and structural support enhance the collective fitness of the bacteriome, complicating host immune recognition and contributing to persistent tissue destruction. Furthermore, by modulating bacterial population densities (78) through density-dependent regulation (often referred to as the ‘kill the winner’ mechanism), phages maintain the high taxonomic diversity characteristic of dysbiotic niches (79–81).

Broadening the taxonomic scope of the subgingival microenvironment, archaea represent a crucial yet often overlooked component. Investigations indicate that the abundance of methanogenic archaea is notably higher in dental plaque collected from periodontally diseased sites compared with periodontally healthy sites, suggesting a positive correlation among archaeal load, periodontal pocket depth, and inflammation severity (82, 83). Specifically, individuals with periodontitis are 6.68 to 9.42 times more likely to harbor subgingival archaea compared with healthy controls (83). Furthermore, while archaea function as secondary colonizers that flourish within the inflammatory environment, they reach a general prevalence of 46% (95% CI: 36–56%) in diseased sites, contrasted with a significantly lower baseline in health of approximately 6.7% (83).

In the periodontal ecosystem, archaea—predominantly methanogenic *Euryarchaeota*—maintain a syntrophic relationship with the bacteriome through interspecies hydrogen transfer (84–86). While anaerobic bacteria ferment proteins and release hydrogen gas as a byproduct, a buildup of this gas inhibits bacterial metabolism (87, 88). Archaea consume this H_2_ to produce methane, thereby alleviating metabolic feedback inhibition (89–91). This synergy optimizes bacterial fermentation and energy production (92), allowing pathogenic bacteria (such as *Porphyromonas gingivalis*) to ferment proteins at an accelerated rate. This accelerated metabolism facilitates the proliferation of periodontal pathogens in deep anaerobic pockets (93, 94) and contributes to increased toxin production and tissue destruction (95). Consequently, the metabolic cooperation provided by archaea indirectly exacerbates host responses and tissue damage by supporting the proliferation of the bacterial community (96–99). Furthermore, archaea may contribute to the pathogenic potential of the subgingival ecosystem by donating virulence-promoting genes to bacteria through horizontal gene transfer (100). These interactions demonstrate that archaea function as modulators of the biofilm, stabilizing and enhancing the dysbiotic structure (89).

Evidence increasingly indicates that host factors—including age, antibiotic exposure, and systemic comorbidities—highly individualize and modulate the oral mycobiome (101–104). Systemic conditions, coupled with the host’s unique genetic susceptibility to viral, fungal, and bacterial challenges, create a permissive environment for synergistic interkingdom interactions. When genetic variations impair the host’s innate sensing and inflammatory regulation, the presence of the archaeome, virome, and mycobiome—though numerically marginal—significantly amplifies bacterial virulence. Therefore, understanding the convergence among local health, systemic health, and the host’s genetic architecture remains essential for developing personalized therapeutic strategies that address the full complexity of the periodontal ecosystem.

### 2.2 Interkingdom crosstalk in periodontitis: a proposed framework for molecular pathogenesis

#### 2.2.1 Bacteria-fungi synergy

Evidence increasingly recognizes complex and often synergistic interactions between bacterial and fungal species as pivotal in the pathogenesis of periodontitis. These interactions largely occur within structured polymicrobial biofilms, where *Candida* and oral streptococci form highly integrated communities (105, 106). In particular, *Candida albicans*, a dimorphic and opportunistic fungus, colonizes the oral cavities of over half the human population, establishing a significant presence that can shift from commensal to pathogenic under certain conditions.

A significant contribution of *Candida albicans* to the polymicrobial environment involves creating conditions favorable for anaerobic bacterial growth. *C. albicans* rapidly proliferates under aerobic conditions, consuming available oxygen and thereby establishing a hypoxic microenvironment within the biofilm. This oxygen depletion remains crucial for the survival and robust growth of obligate anaerobes such as *Porphyromonas gingivalis* in otherwise oxygen-rich oral niches, effectively modifying the microenvironment to facilitate bacterial pathogenicity. Indeed, multiple studies report an increased prevalence and load of *Candida* spp. in periodontal pockets compared with periodontally healthy sites; meta-analytical data derived from 26 clinical studies indicate that the presence of *Candida* spp. is associated with a 1.76-fold increase in the likelihood of developing chronic periodontitis (OR = 1.76; 95% CI: 1.04–2.99). Furthermore, when comparing the biological density of these microorganisms, patients with chronic periodontitis exhibit a significantly higher standardized mean difference in fungal load (SMD = 1.58; 95% CI: 0.15–3.02) compared with periodontally healthy individuals (107). Additionally, *C. albicans* frequently co-locates with key periodontopathogens such as *P. gingivalis*, *Tannerella forsythia*, and *Treponema denticola* in sites affected by periodontitis. The co-isolation of *C. albicans* with *T. forsythia* or *T. denticola* correlates with a greater surface area of inflammation, suggesting a synergistic relationship that exacerbates periodontal tissue damage. Multikingdom analyses suggest that these fungal shifts represent part of a broader multikingdom dysbiosis rather than isolated fungal overgrowth (101, 108).

The interaction between *P. gingivalis* and *C. albicans* involves intricate molecular mechanisms that enhance the overall virulence of polymicrobial biofilms. *C. albicans* promotes the invasion of *P. gingivalis* into human gingival epithelial and fibroblast cells, contributing significantly to tissue destruction (31). Beyond environmental modification, *C. albicans* stimulates the activity of gingipains produced by *P. gingivalis*, which function as critical cysteine proteinases responsible for degrading host proteins and immune mediators. Furthermore, the growth of *C. albicans* via pseudohyphae provides physical scaffolds that facilitate bacterial anchorage during biofilm maturation, significantly increasing bacterial viability and progression on surfaces such as dental implants (10, 109).

Mixed-species biofilms arise through specific adhesion and coaggregation mechanisms. For example, streptococcal cell-surface polysaccharides and adhesins bind to fungal hyphae, thereby mediating stable attachment to bacterial partners (56). The resulting biofilm architecture promotes microbial persistence, spatial organization, and resistance to mechanical clearance. Specific cell wall adhesins—particularly members of the agglutinin-like sequence (Als) family, such as Als3 and Als1—mediate the adherence of *Porphyromonas gingivalis* to *Candida albicans* hyphae. These physical and metabolic interactions collectively enhance the invasive capacity of *P. gingivalis*. Similarly, mixed-species biofilms formed by *C. albicans* and *P. gingivalis* significantly compromise epithelial barrier integrity by disrupting tight junction and adherens proteins, specifically zonula occludens-1 (ZO-1) and E-cadherin (108, 110, 111). A collapse in cellular defense against oxidative stress accompanies these structural shifts, characterized by decreased expression of the cytoprotective enzyme heme oxygenase-1 (HO-1) (111). Furthermore, this environment triggers a shift in apoptotic regulation. Enhanced Bax/Bcl-xL ratios demonstrate this shift, indicating a transition toward pro-apoptotic activity in host cells (111). While *C. albicans* appears to function as a dominant driver of pro-inflammatory responses, *P. gingivalis* contributes through immune modulation and its potent enzymatic activity via gingipains. This intricate interplay highlights that fungal species, particularly *C. albicans*, significantly facilitate bacterial pathogenesis in periodontitis by actively modifying the microenvironment and enhancing bacterial virulence through specific molecular interactions (44). Virulence factors produced by *C. albicans*, such as Secreted Aspartyl Proteases (SAPs) and candidalysin, support this capacity to promote epithelial barrier disruption by actively injuring these tissues (110).

Quorum sensing and interkingdom chemical signaling also play a central regulatory role in bacteria-fungi synergy. Both bacterial and fungal secreted molecules modulate morphogenesis, biofilm maturation, and virulence-associated phenotypes (112). In particular, fungal quorum-sensing molecules such as farnesol influence bacterial behavior, while bacterial signaling molecules alter *C. albicans* filamentation and biofilm dynamics (113, 114). These reciprocal signaling networks allow organisms to sense population density, adapt to local environmental conditions, and coordinate collective behavior within the biofilm (105). Clinically, oral bacterial–fungal biofilms exhibit enhanced tolerance to antimicrobial agents and host immune defenses compared with monospecies biofilms, contributing to chronicity and treatment failure in conditions such as denture stomatitis and other oral mucosal diseases (115). The protective extracellular matrix and altered metabolic states within mixed biofilms reduce drug penetration and efficacy, while coordinated signaling upregulates stress-response and virulence pathways (116).

#### 2.2.2 Bacteria-virus interactions

Herpesviruses promote bacterial proliferation and simultaneously diminish host immune responses in infected oral epithelial cells or macrophages (117). The mutual targeting of oral epithelial cells by both oral viruses and periopathogens creates an environment where distinct types of pathogens thrive and contribute to microbial dysbiosis. Viral introduction or reactivation shifts microbiome diversity, leading to greater ecological imbalance, increased pathogen burden, and compromised oral mucosal immunity. Viral presence exacerbates sustained dysbiosis, severely impairing the intricate interactions between the oral microbiome and oral epithelial cells. This impairment creates an additional risk for novel viruses, such as SARS-CoV-2, to establish a replicative reservoir while simultaneously attenuating host microbial resistance and clearance mechanisms. Pre-existing oral diseases, such as periodontitis, contribute directly and indirectly to oral dysbiosis, increasing the vulnerability of oral mucosal tissues to viral infections and facilitating the reactivation of latent oral viruses (118).

Shared adhesion and entry mechanisms represent a significant aspect of interkingdom synergy. Oral bacterial and viral pathogens often exploit common pathways to enter host cells, increasing the susceptibility of the oral cavity to infection. For example, heparan sulfate proteoglycans (HSPG) serve as attachment receptors for both DNA and RNA viruses, facilitating entry into epithelial cells (119). Herpes simplex virus envelope glycoproteins B and C bind to HSPG, mediating viral attachment. Furthermore, enveloped viruses exploit host cell molecules through a process known as apoptotic mimicry. These viruses induce the externalization of phosphatidylserine on host cells, acquire this phosphatidylserine, and incorporate the molecule into their viral membranes. By structurally resembling apoptotic bodies, viruses enter host cells via the phosphatidylserine receptor (120). This strategy functions as an immune evasion tactic and exacerbates inflammatory outcomes by inducing an anti-inflammatory response and dampening pro-inflammatory cytokine production, thereby weakening oral immunity (118).

Periodontal pathogens also contribute through enzymatic assistance; specifically, *Streptococcus gordonii* releases a metallo-serine endopeptidase that mimics furin. Enzymatic activity from this bacterium assists oral viruses, such as human papillomavirus, by activating the HSPG receptor (121). These molecular mechanisms demonstrate that oral viruses significantly contribute to periodontitis pathogenesis by actively modulating host immunity and facilitating bacterial colonization and virulence. Viral agents weaken host defenses and open pathways that facilitate bacterial invasion and proliferation (117).

#### 2.2.3 Bacteria-archaea symbiosis

Archaea represent a distinct domain of life within the subgingival microbiome, with methanogenic phylotypes such as *Methanobrevibacter oralis*, *M. smithii*, *M. massiliense*, and *Methanomassiliicoccus luminyensis* constituting the predominant inhabitants (95). While evidence does not typically classify archaea as primary pathogens, these microorganisms significantly modulate metabolism within the periodontal pocket. Historically, researchers identified the previously described hydrogenotrophic metabolism as the primary mechanism driving this contribution (89). However, moving beyond this classical model, current perspectives on the multikingdom phylogeny of the oral microbiome emphasize that bacteria-archaea symbiosis relies on sophisticated metabolic handoffs and direct physical interactions. These relationships often involve Direct Interspecies Electron Transfer (DIET) (122), where partners exchange energy via conductive cellular structures rather than through the diffusion of soluble gases (122). Concepts such as “leaky” metabolisms and the Black Queen Hypothesis further solidify this cooperation, wherein evolutionary pressure leads to a distributed metabolic network; archaea and bacteria become obligate partners not just for carbon cycling, but for the exchange of essential vitamins and amino acids that neither organism can synthesize in isolation.

Within the structural complexity of biofilms, this symbiosis facilitates niche construction and community-wide homeostasis. Evidence suggests that archaea contribute to stability within low-energy microenvironments by scavenging potentially toxic methylated byproducts generated by bacterial fermentation (123, 124). This metabolic scavenging reduces niche toxicity, allowing a diverse phylogenetic landscape to persist. Ultimately, this interkingdom synergy provides ecological protection; by occupying specific functional guilds, these stable consortia inhibit the colonization of opportunistic pathogens, ensuring the resilience and stability of the host-associated ecosystem (125). These emerging perspectives suggest that the role of archaea in the periodontal pocket extends into a more complex regulatory capacity than previously hypothesized. Rather than acting solely as terminal hydrogen consumers, these microorganisms facilitate the exchange of specialized metabolites and electrons. This multikingdom arrangement stabilizes the subgingival environment, making the biofilm more resilient to host-derived oxidative stress or nutrient fluctuations. Consequently, the pathogenicity of the subgingival microbiota does not function solely through individual ‘red complex’ bacteria (belonging to the Bacteroidota and Spirochaetota phyla), but rather emerges from a highly interconnected, cross-domain network (126–128).

#### 2.2.4 Phage-bacteria dynamics

Although bacteriophages represent a substantial segment of the periodontal landscape, a comprehensive understanding of their specific ecological roles and regulatory impacts remains an emerging frontier in oral microbiology. Phage infection of periodontopathogens, such as *Fusobacterium nucleatum*, alters bacterial metabolic pathways to produce tryptophan metabolites, including indole (129). These indole metabolites act as agonists of the host aryl hydrocarbon receptor, triggering signaling cascades that result in immunosuppression and reduced viral clearance (130). Building on their previously described role in horizontal gene transfer during early dysbiosis, lysogenic conversions—in which integrated phages confer new traits to their bacterial hosts—provide selective advantages in response to antibiotics or other environmental disturbances. This process demonstrates that phages function not merely as regulators of bacterial populations, but as active participants in bacterial evolution and adaptation within the dysbiotic environment.

Preliminary observations suggest that phages contribute to the structural dynamics of the periodontal microbial landscape, facilitating the transition from health to periodontitis (62). Complex life-history strategies, such as the lysogenic-to-lytic switch, frequently mediate this regulation and alter community composition in response to environmental stressors. Studies in other ecosystems, such as the human gut (131), indicate that prophages impose fitness trade-offs on their bacterial hosts while simultaneously enhancing host competitiveness against rival strains. In the context of the periodontal pocket, this dynamic implies that certain pathobionts achieve ecological dominance through phage-encoded traits or phage-mediated competitive exclusion. Given this regulatory influence, evidence demonstrates a growing interest in utilizing bacteriophages for targeted periodontal pathogen control, a strategy increasingly explored in recent systematic assessments (132, 133).

The application of advanced sequencing technologies has significantly refined our interpretation of these interactions. Long-read metagenomics uncovers high-resolution host-virus associations that were previously invisible, revealing a highly specific interaction network between oral phages and their bacterial targets (134). These findings mirror observations in the urinary tract (135) and human gut (74) microbiomes, where phages act as community modulators that maintain microbial homeostasis or, conversely, contribute to dysbiotic shifts. The implications of these phage communities extend to host health and disease (136); in periodontitis, a shift in the virome frequently represents an early indicator of ecological imbalance (136), where viral predation or horizontal gene transfer facilitates the emergence of virulent bacterial lineages within the increasingly unstable subgingival environment. However, the full characterization of the oral virome remains constrained by a high proportion of uncharacterized sequences that lack taxonomic assignment, a limitation compounded by the significant bioinformatic challenges of achieving precise *in vivo* phage-host matching within complex, multi-kingdom biofilms.

### 2.3 Systems-level analysis of interkingdom crosstalk: an omics perspective

The integration of high-throughput multi-omics technologies has largely catalyzed the shift toward a systems-level understanding of the subgingival microbiome. Whole-metagenome shotgun sequencing (WMS) provides the essential technical foundation for this progress, allowing for an unbiased analysis of complex communities that traditional culture-dependent methods failed to capture (137). Applying integrative omics approaches to fungi, archaea, and viruses reveals that these frequently overlooked microorganisms function as active participants in the subgingival ecosystem rather than transient colonizers (94). While metagenomics effectively bypasses the limitations of cultivation to enable an expansive exploration of this viral, bacterial, fungal, and archaeal sequence space, complementary approaches such as metatranscriptomics and metabolomics are now required to map active gene expression and complex metabolic handoffs across kingdoms. Despite these advances, the systems-level analysis of interkingdom crosstalk faces ongoing limitations, including the reliance on incomplete reference databases for non-bacterial microorganisms and the significant computational challenges of integrating heterogeneous, multikingdom datasets. However, the lack of a standardized taxonomic framework across all kingdoms represents a significant challenge in moving toward a holistic model. While the 16S ribosomal RNA (rRNA) universal marker gene facilitates the development of robust repositories—such as the Human Oral Microbiome Database (HOMD), SILVA, Greengenes, and the Ribosomal Database Project (RDP)—other microbial community members lack a comparable universal anchor. For instance, mycobiome analysis often relies on the highly variable internal transcribed spacer (ITS) region, whereas repositories such as SILVA, Greengenes, and the Genome Taxonomy Database (GTDB) document the archaeome with varying levels of coverage.

This lack of a common marker is most acute in the virome and phageome, where high mutation rates necessitate a reliance on WMS to reconstruct viral genomes. The loss of contextual information, specifically host-virus associations, represents a significant challenge in such metagenome-derived data (138). Integrated machine-learning frameworks, such as iPHoP, address this challenge by utilizing gene-sharing networks (a concept validated by systems biology (139) for the taxonomic assignment of uncultivated viruses. Combining these networks with extensive ecological metadata from databases such as IMG/VR v4 (140), RefSeq, Prokaryotic Virus Orthologous Groups (pVOGs), and International Committee on Taxonomy of Viruses (ICTV) taxonomies enables researchers to move beyond simple gene lists toward a functional understanding of how these sequences operate within specific human niches. These computational advancements indicate, for example, that viruses—including prophages integrated into bacterial DNA and documented in specialized resources like the Gut Phage Database (GPD) or PhageDB—function as significant modulators of interkingdom crosstalk. Through horizontal gene transfer or selective predation, these viral components alter the metabolic trajectories of both bacterial and archaeal hosts.

Beyond taxonomic identification, the transition from metagenomics to metatranscriptomics and metabolomics enables researchers to interpret the functional state of the microbiome rather than merely its genetic potential. While metagenomics delineates the genetic composition of the community, metatranscriptomics captures the active gene expression patterns that define real-time responses to the periodontal environment, providing a crucial perspective for identifying the primary drivers of microbial dysbiosis (94). This functional layering remains vital for observing the previously discussed metabolic handoffs; for instance, the active expression of archaeal methanogenesis genes in tandem with bacterial fermentative pathways provides a robust indication of syntrophic synergy. When coupled with metabolomics—the direct measurement of chemical outputs such as methylated compounds or signaling molecules—these technologies advance the field toward a holistic model. Within this framework, interkingdom interactions represent emergent properties of a highly coordinated, multi-omic landscape that modulates the transition between microbial homeostasis and the inflammatory shifts characteristic of periodontitis.

## 3 Variations in microbial diversity and composition in health vs. disease severity

Research into the subgingival microbial composition has revealed significant variations in microbial diversity and composition associated with different states of periodontal health and varying degrees of periodontitis severity. Deeper periodontal pockets (probing depths [PD] of 6–9 mm) exhibit significantly higher microbial richness and overall diversity compared with periodontally healthy sites (PD ≤3 mm). This difference indicates that deeper pockets provide diverse, anaerobic-friendly environments that support a broader and more balanced, yet highly dysbiotic, microbial community (141).

At the phylum level, Proteobacteria decrease in relative abundance from periodontally healthy to diseased states, whereas phyla such as Saccharibacteria (TM7), Spirochaetes, Synergistetes, Chloroflexi, and Tenericutes demonstrate increasing trends across periodontitis stages. Specifically, the deepest pockets (probing depths [PD] of 6–9 mm) exhibit a significantly higher relative abundance of Saccharibacteria (TM7) compared with periodontally healthy sites (142).

The shifts at the genus level are even more pronounced. Genera such as *Fusobacterium*, *Prevotella*, *Treponema*, and *Tannerella* increase in relative abundance from health to periodontitis, indicating their elevated presence in inflamed periodontal sites. Conversely, genera typically associated with health, such as *Streptococcus*, *Leptotrichia*, *Neisseria*, and *Corynebacterium*, demonstrate decreasing trends. Notably, *Corynebacterium* and *Cardiobacterium* decrease, while *Schaalia* increases in physiologic gingival sulci or gingivitis-associated sites (PD ≤3 mm) compared with periodontally healthy controls. These shifts indicate early microbial alterations or subclinical dysbiosis even in sites that appear clinically stable or lack deep pocket formation, yet may already harbor clinical attachment loss (37).

The progression of periodontitis follows a predictable ecological succession of microbial communities, reflecting the polymicrobial synergy and dysbiosis (PSD) model (142). The ‘red complex’ species—including *Porphyromonas gingivalis* and *Tannerella forsythia* (belonging to the Bacteroidota phylum), alongside *Treponema denticola* (belonging to the Spirochaetota phylum)—predominantly colonize deeper pockets (probing depths [PD] of 4–5 mm and 6–9 mm). These species exhibit strong positive correlations among themselves, reflecting their synergistic roles in advanced disease (4). Furthermore, ‘orange complex’ species, such as *Fusobacterium nucleatum* (belonging to the Fusobacteriota phylum), appear earlier in disease progression. These species function as structural coaggregation bridges, facilitating the colonization of both early- and late-colonizing pathogens.

Despite the broad characterization of bacterial diversity and composition differences across periodontal health and disease severity, evidence demonstrates that non-bacterial microbial kingdoms also exhibit clinically relevant alterations along the health–periodontitis continuum. These interkingdom changes mirror disease severity and contribute to progressive derangements in host–microbiome balance. The oral mycobiome in periodontal health is mainly characterized by low fungal biomass, low diversity, and Candida species functioning as commensals in a yeast-dominant state. As periodontal inflammation progresses, the load and diversity of fungal species increase, especially in deeper periodontal pockets, where Candida albicans thrives and shifts toward hyphal growth or biofilm-driven phenotypes (101, 143). Clinical and sequencing studies report that the presence and prevalence of Candida increase linearly with probing depth and clinical attachment loss, indicating that fungal enrichment correlates with disease severity, rather than mere presence (108). Most pertinently, fungal dysbiosis appears to be a feature of interkingdom dysbiosis, as communities with more advanced periodontal damage and greater bacterial colonization exhibit the greatest fungal overrepresentation (101). Conversely, periodontitis, particularly its severe manifestations, correlates with the heightened detection and reactivation of human viruses, most notably herpesviruses such as Epstein–Barr virus, cytomegalovirus, and herpes simplex virus type 1 (60, 117). Studies demonstrate greater viral virulence and diversity in deeper periodontal pockets compared with periodontally healthy sites, where viral presence positively associates with probing depth, bleeding on probing, and overall disease severity (31, 144–146).

While archaea represent a numerically minor fraction of the subgingival microbiome—shifting from nearly undetectable levels in periodontally healthy sites to approximately 1%–5% of the total microbial load in periodontitis (83)—their presence strongly correlates with dysbiotic sites. This significant increase in both prevalence and relative abundance indicates that methanogens function as integral components of the pathogenic consortium, facilitating the metabolic activity of more dominant bacterial species.

Clinical prevalence data underscore the contribution of the archaeome to periodontal dysbiosis; specifically, the targeted detection of *Methanobrevibacter oralis* reveals a significant disparity, with prevalence rates rising from 6.7% in periodontally healthy individuals to 40% in subjects with chronic periodontitis (83). This notable increase in detection frequency within deeper periodontal pockets indicates a strong association between methanogenic archaea and the localized ecological shifts that characterize chronic periodontitis (95, 99). Furthermore, quantitative studies demonstrate that archaeal abundance correlates with probing depth and the anaerobicity of the periodontal pocket, affirming that colonization represents a late ecological event associated with advanced disease stages (89). Consequently, archaeal enrichment reflects progressively greater metabolic complexity and the ecological maturation of dysbiotic biofilms, rather than early disease initiation.

Within periodontally healthy sites, the phageome primarily comprises lytic bacteriophages, which regulate bacterial community structure and inhibit the growth of pathogenic taxa. During the early transition toward dysbiosis, these viral dynamics shift gradually; consequently, as disease severity increases, lysogenic phage communities incorporated into bacterial genomes become increasingly prevalent, particularly in advanced periodontitis (59, 62). High phage diversity and prophage abundance in deeper periodontal pockets demonstrate an integrated, pathogenic microbial system associated with disease severity (62). Collectively, these findings indicate that the severity of periodontal disease correlates with interkingdom restructuring rather than independent bacterial reprogramming. Periodontally healthy sites exhibit low fungal and archaeal abundance (0.1%) (83), a bacterial community regulated predominantly by lytic phages, and low overall viral activity. Conversely, severe periodontitis demonstrates a higher fungal and viral load (with Epstein–Barr virus [EBV] DNA detected in 37.3% of periodontitis cases) (147), an enrichment of methanogenic archaea, and a phageome skewed toward lysogeny. These shifts indicate a highly complex and robust dysbiotic ecosystem. Ultimately, such coordinated interkingdom transitions demonstrate that periodontitis severity mirrors enhanced microbial diversity, functional complexity, and pathogenic potential across various microbial kingdoms.

This detailed understanding of microbial shifts demonstrates that the subgingival environment functions as an active ecological niche that shapes the composition and intricate interkingdom interactions of the resident microbiome, facilitating the development of a more complex and pathogenic community in deeper periodontal pockets (10). **Table 1** summarizes key microbial species and their interkingdom associations in periodontitis.

**Table 1:** Key microbial species in periodontitis and their interkingdom associations.

Kingdom	Representative species	Typical location/role in health/disease	Known interkingdom partners/synergistic effects	Key virulence factors/mechanisms
Bacteria	*Porphyromonas gingivalis*	Subgingival pathogen, keystone pathogen	*Candida albicans, Herpesviruses, Treponema denticola, Tannerella forsythia*	Gingipains, LPS, fimbriae, OMVs, capsule
	*Tannerella forsythia*	Subgingival pathogen, red complex	*P. gingivalis, T. denticola, C. albicans*	S-layer glycoproteins, dipeptidyl aminopeptidase IV
	*Treponema denticola*	Subgingival pathogen, red complex	*P. gingivalis, T. forsythia, C. albicans*	Dentilisin, outer sheath proteins, internal flagella
	*Aggregatibacter actinomycetemcomitans*	Subgingival pathogen, linked to aggressive periodontitis	Herpesviruses	Leukotoxin (LtxA), cytolethal distending toxin (CDT), tad genes for biofilm
	*Fusobacterium nucleatum*	Bridge bacterium, early/late colonizer	Orange complex, TM7 phylum	Adhesins (FadA), LPS, serine protease
	*Prevotella* spp*. (P. intermedia)*	Subgingival pathogen, obligate anaerobe	–	Adhesion, hemolysin, hemagglutinin, proteolytic enzymes
Fungi	*Candida albicans*	Commensal, opportunistic pathogen	*P. gingivalis, S. mutans, S. aureus*	Dimorphism (yeast/hyphae), Als adhesins, oxygen consumption, aspartyl proteases
Viruses	Herpesviruses (HSV-1, EBV, CMV)	Oral mucosal lesions, gingival inflammation	Bacterial periopathogens (e.g., *Porphyromonas gingivalis*, *Aggregatibacter actinomycetemcomitans*)	Shared adhesion receptors (HSPG), apoptotic mimicry (PS externalization), immune modulation
Archaea	*Methanobrevibacter oralis*	Subgingival, methanogenic phylotype	Hydrogen-producing bacteria (syntrophic partners)	Hydrogenotrophic metabolism, methane production, potential HGT donor
Phages	Bacteriophages	Abundant in oral microbiome	Bacterial hosts	Lysis, lysogeny, horizontal gene transfer (virulence/resistance genes), influence host metabolism

LPS, Lipopolysaccharide; OMVs, Outer Membrane Vesicles; HSV-1, Herpes Simplex Virus Type 1; EBV, Epstein-Barr Virus; CMV, Cytomegalovirus; HSPG, Heparan Sulfate Proteoglycans; PS, Phosphatidylserine; HGT, Horizontal Gene Transfer.

## 4 Systemic implications of interkingdom interactions in periodontitis

Periodontitis is not merely a localized oral disease; it demonstrates profound systemic effects, contributing to a wide array of non-communicable diseases (NCDs) throughout the body. This broad impact correlates with the complex interkingdom interactions within the oral microbiome and the subsequent systemic inflammatory burden. **Table 2** summarizes the most relevant mechanisms of interkingdom crosstalk and their systemic interactions in humans.

**Table 2:** Potential interkingdom players involved in the oral-systemic health axis.

Systemic condition/interkingdome role	Archaea (metabolic facilitator)	Mycobiome (structural scaffold)	Virome (genetic/immune modulator)	Primary systemic impact
Cardiovascular (CVD)	Enhances gingipain production via H_2_ sink	Functions as a shield for arterial transport, protecting bacterial aggregates from shear stress during transit	Extensive lysis releases LPS, promoting endothelial inflammation	Increased systemic toxin levels promote atherosclerotic plaque instability and thrombosis
Diabetes (T2DM)	Increases metabolic waste (ammonia), which modulates hepatic function	Feeds on high GCF glucose, thickening the biofilm scaffold	Interference with the insulin receptor triggers TLR4-mediated inflammation, worsening insulin resistance	Metabolic endotoxemia and impaired glucose homeostasis.
IBD (Crohn's/UC)	Modifies local pH and energy efficiency for gut colonization.	Shields oral pathogens from stomach acid during swallowing.	Genetic reprogramming of gut flora by transferring virulence genes between oral and gut species.	Ectopic gut colonization and chronic intestinal dysbiosis.
Alzheimer's	Accelerates neurotoxic protease (gingipain) secretion.	Provides structural protection facilitating bacterial transit across the BBB.	Direct microglia activation via released bacterial DNA/toxins.	Neuroinflammation and amyloid-beta accumulation.
RA / Autoimmunity	Maximizes hyper-citrullination rates by *Porphyromonas gingivalis*	Acts as an adjuvant, amplifying the local immune response.	Modifies bacterial genomes to express highly immunogenic epitopes	Production of Anti-Citrullinated Protein Antibodies (ACPAs).
Pregnancy Outcomes	Increases total pathogen load in the oral reservoir.	Facilitates invasion of the bloodstream toward the placenta.	Promotes localized inflammatory responses	Preterm birth, preeclampsia, and low birth weight.

CVD, Cardiovascular Disease; H_2_, Hydrogen gas; LPS, Lipopolysaccharide; T2DM, Type 2 Diabetes Mellitus; GCF, Gingival Crevicular Fluid; TLR4, Toll-Like Receptor 4; IBD, Inflammatory Bowel Disease (including Crohn's Disease and Ulcerative Colitis); BBB, Blood-Brain Barrier; RA, Rheumatoid Arthritis; ACPAs, Anti-Citrullinated Protein Antibodies.

Although both bacterial dissemination and endotoxemia (low-grade, acute, or chronic systemic inflammation triggered by circulating bacterial endotoxins) have historically been proposed as the primary determinants of systemic associations, increasing evidence demonstrates that microbial contributions to the systemic inflammatory burden also arise from non-bacterial members of the oral microbiome. These interkingdom components actively modulate host immune responses, metabolic pathways, and immune tolerance systems. For example, Fungal components of the oral microbiome, particularly *Candida albicans*, contribute to systemic inflammation not only by inducing local tissue injury but also through sustained immune activation. Chronic oral fungal colonization correlates with elevated levels of pro-inflammatory cytokines—such as Interleukin-6 (IL-6), Interleukin-17 (IL-17), and Tumor Necrosis Factor-alpha (TNF-α)—which function as important mediators of cardiometabolic diseases and immune processes (44). Notably, fungal-driven Th17 polarization, which is typically characteristic of mucosal immunity, spreads systemically and subsequently promotes endothelial dysfunction, insulin resistance, and disturbances in bone remodeling. In this context, the enrichment of fungi within polymicrobial biofilms in periodontitis functions as a chronic immunogenically stimulatory agent. This enrichment lowers the threshold for developing systemic inflammatory diseases by maintaining a low-grade, chronic inflammatory state (108). Oral viruses, particularly herpesviruses, contribute to severe systemic effects not merely by disseminating throughout the host via direct transmission, but also by actively modifying host immune competence. Viral reactivation within periodontal tissues affects interferon signaling, dysregulates macrophage and dendritic cell responses, and alters T-cell polarity (117). These immune-modulatory mechanisms propagate systemically, rendering the host susceptible to exaggerated inflammatory responses to external stimuli. This viral-mediated immune dysregulatory process provides a mechanistic pathway linking periodontitis to systemic diseases characterized by immune dysregulation, such as cardiovascular and metabolic diseases. Consequently, oral viruses function as system-wide amplifiers of inflammatory susceptibility; in combination with weakened immune surveillance, they serve as mediators of systemic disease rather than functioning solely as site-specific local pathogens (148). Methanogenic archaea contribute to systemic disease risk by maintaining metabolically active dysbiotic communities. By facilitating anaerobic bacterial metabolism, archaea indirectly contribute to the continuous release of microbial metabolites and inflammatory mediators into the systemic circulation (89). This steady metabolic output promotes immune activation and modulates systemic mechanisms implicated in insulin resistance, lipid metabolism, and bone homeostasis. Although archaea themselves do not typically elicit direct immune responses, their stabilization of highly inflammatory microbial ecosystems makes them important indirect contributors to the chronic, low-grade systemic inflammation associated with periodontitis (95, 149).

### 4.1 Periodontitis and cardiovascular diseases

A robust and independent association exists between periodontitis and various cardiovascular diseases, including coronary, peripheral, and cerebrovascular conditions, as well as acute coronary events such as myocardial infarction (150). Periodontal disease promotes systemic inflammation through bacteremia (direct bacterial dissemination) and the release of pro-inflammatory mediators, including C-reactive protein (CRP), matrix metalloproteinases (MMPs), Interleukin-1β (IL-1β), Interleukin-6 (IL-6), and Tumor Necrosis Factor-alpha (TNF-α). This sustained systemic inflammatory burden negatively impacts the development and progression of cardiovascular conditions (151).

Periodontal pathogens (including *Porphyromonas gingivalis*, *Aggregatibacter actinomycetemcomitans*, *Tannerella forsythia*, and *Streptococcus mutans*) enter the bloodstream through inflamed gingival tissues. Once in circulation, these bacteria have been identified in atherosclerotic lesions and in aspirated thrombi from patients with acute ischemic stroke, where they penetrate arterial walls to promote inflammation, plaque disruption, and clot formation (152). Beyond direct bacterial presence, pathogenic mechanisms involving lipopolysaccharides (LPS) from Gram-negative bacteria induce a sustained immune reaction at the vascular and endothelial levels. This reaction elicits systemic host immune responses and alters the barrier function of the vascular endothelium, thereby facilitating further pathogen penetration (153).

Oxidative stress generated by reactive oxygen species (ROS) during periodontal infection and inflammation contributes to cellular and extracellular matrix dysfunction, DNA damage, and altered lipid metabolism, all of which are implicated in the development of cardiovascular disease (154). Pathogenic oral bacteria also produce harmful metabolites, such as trimethylamine N-oxide (TMAO), which negatively impact cardiovascular health (155). Specifically, TMAO promotes systemic pathology by upregulating the expression of scavenger receptors (CD36 and SR-A1) on macrophages and inhibiting reverse cholesterol transport, thereby facilitating foam cell formation and arterial plaque accumulation. This metabolic disruption is further exacerbated by the inhibition of the hepatic enzymes CYP7A1 and CYP27A1, which suppresses the body’s primary route for cholesterol elimination via bile acid synthesis. At the vascular level, TMAO activates the Mitogen-Activated Protein Kinase (MAPK) and Nuclear Factor kappa B (NF-κB) inflammatory signaling pathways. This activation increases the expression of adhesion molecules such as Vascular Cell Adhesion Molecule-1 (VCAM-1) and Intercellular Adhesion Molecule-1 (ICAM-1), while simultaneously inducing further ROS production and inhibiting endothelial nitric oxide synthase (eNOS) to compromise vasodilation. Furthermore, elevated TMAO levels correlate with increased intestinal and epithelial permeability, facilitating the translocation of microbial products into the bloodstream and contributing to chronic systemic endotoxemia (156–159). This multifaceted process—involving systemic inflammation, direct microbial invasion, and the production of pro-atherogenic metabolites—demonstrates how interkingdom dysbiosis in periodontitis contributes to the pathogenesis of distant systemic diseases (160).

The multi-kingdom perspective prompts a fundamental reevaluation of the mechanistic link between periodontitis and cardiovascular disease (CVD), transitioning from a model of transient bacteremia to one of coordinated systemic virulence networks. As previously described, methanogenic archaea function as a hydrogen sink within the subgingival niche, facilitating interspecies hydrogen transfer and optimizing the bioenergetics of the surrounding anaerobic consortia (91). By accelerating the fermentative metabolism of proteinaceous substrates by *Porphyromonas gingivalis* and other asaccharolytic pathogens, these archaea upregulate the synthesis and secretion of cysteine proteases, specifically gingipains (91). These enzymes transition into the systemic circulation, where they contribute to the proteolysis of plasma proteins, disrupt the coagulation cascade, and promote the structural instability of atherosclerotic plaques (12). Consequently, by catalyzing bacterial metabolic flux, archaea increase the circulating concentration and systemic bioavailability of these microbial toxins.

Regarding the mycobiome, current hypotheses propose that polymicrobial biofilms—composed of synergistic bacterial and fungal communities—can dislodge from periodontal pockets as integrated structural entities (115, 161, 162). These structures exhibit enhanced resistance to both antimicrobial agents and the shear stress of blood flow (163, 164), thereby facilitating the transport of intact, viable bacterial and fungal cells to the coronary arteries (165).

The virome contributes two critical mechanisms to this relationship. First, extensive phage-mediated bacterial lysis facilitates the chronic release of high levels of lipopolysaccharides (LPS) and bacterial DNA. This continuous release contributes to a state of metabolic endotoxemia, thereby promoting endothelial inflammation—a critical initial step in atherosclerosis (66). Second, as previously described, phages transfer resistance and virulence genes between oral bacteria via horizontal gene transfer. This genetic exchange promotes the emergence of highly virulent bacterial strains with an enhanced capacity for endothelial invasion (166).

Furthermore, recent evidence indicates a multi-kingdom synergy within the atheromatous plaque itself, as atherosclerotic plaques contain not only bacterial DNA but also fungal and archaeal DNA (167). Thus, the mechanistic link extends beyond isolated bacterial dissemination; it involves a complete dysbiotic ecosystem interacting with the host immune system. The pronounced inflammatory response—promoted by phages, fungi, and archaea-accelerated metabolism—contributes to a persistent, low-grade systemic inflammation. This chronic inflammatory state functions as a key mediator of cardiovascular risk in patients with periodontitis (16, 168).

### 4.2 Periodontitis and diabetes

A significant and well-documented bidirectional relationship exists between periodontitis and diabetes, characterized by a reciprocal pathological cycle in which each condition exacerbates the other (169). In patients with diabetes, hyperglycemia promotes the formation of advanced glycation end products (AGEs) and increases oxidative stress (170). Elevated glucose levels modulate the Receptor Activator of Nuclear Factor Kappa-B Ligand to Osteoprotegerin (RANKL/OPG) ratio, contributing to immune cell dysfunction, cytokine imbalance, and altered bone homeostasis, which collectively accelerate periodontal destruction (171). Conversely, the chronic inflammation and microbial dysbiosis associated with periodontitis aggravate glycemic control in individuals with diabetes (172).

The practical implications of this bidirectional relationship are substantial for clinical management, as periodontal treatment can modestly improve levels of circulating mediators related to glycemic control in patients with diabetes (173). Reducing the burden of proinflammatory proteins, cytokines, bacteria, and bacterial antigens through periodontal therapy attenuates systemic inflammation; consequently, this reduction can enhance insulin signaling and decrease insulin resistance. This evidence indicates that addressing oral health provides a therapeutic intervention capable of positively influencing systemic status. Ultimately, the management of periodontitis—by targeting interkingdom dysbiosis and chronic inflammation—offers a viable pathway to mitigate systemic conditions such as diabetes and highlights the critical importance of integrated medical-dental care (174).

While the fundamental multi-kingdom mechanisms previously described remain consistent, the biological destinations and specific inflammatory pathways differ. In the relationship between periodontitis and Type 2 Diabetes Mellitus (T2DM) or other metabolic diseases, this multi-kingdom network functions as a metabolic disruptor rather than merely a localized source of tissue damage. The acceleration of bacterial metabolism by archaea promotes the production of high levels of short-chain fatty acids (SCFAs) and ammonia (89, 91). Although physiological levels of SCFAs are beneficial, their excessive accumulation in the systemic circulation interferes with insulin signaling in the liver and adipose tissue (175). Furthermore, extensive phage-mediated bacterial lysis releases significant amounts of LPS into the systemic circulation (66). In patients with diabetes, this activates Toll-Like Receptor 4 (TLR4) not only in blood vessels but also in metabolic tissues, contributing to a state of chronic, low-grade systemic inflammation (176). This process, known as metabolic endotoxemia, ultimately impairs cellular responsiveness to insulin (177). Conversely, diabetes elevates glucose levels in the gingival crevicular fluid, providing an abundant nutrient source for the mycobiome (178–180). This dynamic establishes a self-perpetuating cycle in which diabetes promotes the expansion of these fungal structural scaffolds, thereby rendering the periodontal biofilm increasingly resistant to antimicrobial treatment.

### 4.3 Oral-gut axis and inflammatory bowel disease

A growing body of evidence indicates a bidirectional relationship between oral pathologies, particularly periodontitis, and inflammatory bowel disease (IBD). This connection prompts a fundamental shift from viewing the two conditions as independent diseases to understanding them as components of a complex, reciprocal cycle (181). The oral cavity, characterized by a unique interkingdom microbiome, serves as a reservoir for pathogens and inflammatory mediators that contribute to dysbiosis and inflammation at distant sites, such as the gut (181). Oral microorganisms and their associated metabolic byproducts reach the gastrointestinal tract primarily through ingestion or systemic circulation. Although gastric acidity typically inactivates a high percentage of swallowed oral microbes, compromised host defense mechanisms—resulting from genetics, systemic disorders, medications, lifestyle factors, or aging—allow these microorganisms to colonize the intestine and promote aberrant immune responses (182).

The pathogenesis of both periodontitis and IBD involves a complex interplay between host immunity and specific bacterial stimuli, characterized by shared roles for various immune cells, including neutrophils, macrophages, dendritic cells, T cells, and B cells (183). This shared immunoinflammatory pathway indicates a common underlying inflammatory predisposition or response (184). The multi-hit hypothesis for periodontitis-mediated IBD pathogenesis proposes a specific sequence of events: an initial dysbiosis of the oral microbiota, followed by a disrupted intestinal barrier, ultimately promoting an aberrant immune response within the gut (185). This sequence illustrates how interkingdom dysbiosis in the oral cavity contributes to systemic diseases by colonizing distant microbiomes and activating shared inflammatory pathways (186).

The connection between periodontitis and IBD from an interkingdom perspective centers on ectopic colonization—the concept of the oral cavity functioning as a reservoir for gut pathogens (187, 188). In the context of IBD, the primary challenge for oral pathogens is surviving the acidic gastric environment to reach the intestines (182, 189). Here, the fungal scaffold (mycobiome) functions as a protective physical barrier, facilitating the survival of oral bacterial-fungal aggregates during transit to the gut (190).

Simultaneously, the virome functions as a key mediator of gut dysbiosis. This viral influence facilitates the transition from a commensal gut microbiome to an inflammatory profile (191, 192). The resulting synergistic biofilm in the gut—specifically the interaction between *Escherichia coli* and *Candida* spp.—is a recognized hallmark of Crohn’s disease (193). The multi-kingdom perspective indicates that these pathogenic biofilms originate from, or are reinforced by, the chronic ingestion of oral polymicrobial units.

### 4.4 Other systemic associations

Periodontitis is associated with over 50 systemic conditions, underscoring the broad impact of the disease on overall health. A recurring mechanistic theme across these associations is the contribution of oral interkingdom dysbiosis to a chronic systemic inflammatory burden.

Chronic periodontitis is associated with various cancers, including head and neck, colorectal, breast, and pancreatic malignancies (194). Persistent infection and inflammation from periodontal disease contribute to chronic systemic inflammation, thereby modulating pro-oncogenic pathways (195). The oral microbiome facilitates the establishment and progression of potentially malignant disorders; for example, by contributing to ethanol metabolism and the formation of acetaldehyde, a process that increases the risk of head and neck cancers (196). Specific bacterial species, such as *Fusobacterium nucleatum* and *Porphyromonas gingivalis*, are associated with oral squamous cell carcinoma pathogenesis through mechanisms that include epithelial cell transformation and enhanced invasiveness (197).

Periodontal disease and associated bacteremia are associated with adverse effects on reproductive health and fertility in both men and women. The condition correlates with adverse pregnancy outcomes such as preterm birth, preeclampsia, miscarriage, and low birth weight (184). Subclinical infections associated with periodontitis impair reproductive function, while hormonal shifts during pregnancy exacerbate existing periodontal disease. Furthermore, the systemic dissemination of endotoxins and inflammatory mediators from periodontal bacteria promotes an inflammatory response within the fetoplacental unit, thereby modulating fetal growth and contributing to obstetric complications (184).

Systemic inflammation also serves as a key mechanistic link between periodontitis and neurological conditions such as Alzheimer’s disease (160). Chronic systemic infection with *Porphyromonas gingivalis* promotes β-amyloid accumulation in the brain and activates inflammatory monocytes and macrophages. Furthermore, elevated proinflammatory cytokines are observed in older patients with both Alzheimer’s disease and periodontitis, and microbial toxins and antibodies derived from periodontal pathogens have been isolated from the brains of patients with Alzheimer’s disease (198).

Microorganisms residing in periodontal pockets, particularly anaerobic bacteria, are aspirated into the lower airways (199). These aspirated microbes exacerbate the local inflammatory burden within lung tissues, thereby contributing to respiratory conditions such as pneumonia, asthma, and chronic obstructive pulmonary disease (COPD).

## 5 Therapeutic strategies: a polymicrobial and interkingdom approach

### 5.1 Limitations of traditional therapies

Traditional approaches to periodontitis treatment rely primarily on mechanical debridement, such as scaling and root planing, to reduce the bacterial load on affected root surfaces (200). While mechanical debridement proves effective in many cases, the procedure represents a non-specific form of treatment for what is now understood to be a complex, polymicrobial, and dysbiotic disease (108). A significant limitation of adjunctive antimicrobial therapies is that they typically target only a single causative agent. This restriction frequently contributes to reduced efficacy and an increased risk of treatment failure when clinicians address complex interkingdom polymicrobial infections (201). Furthermore, an exclusive therapeutic focus on the bacterial compartment inadvertently contributes to the broader challenge of antimicrobial resistance. Therapies targeting a single kingdom possess inherent limitations because periodontal pathogenesis is driven not solely by the presence of individual pathogens, but by the intricate synergistic interactions among diverse microorganisms (202).

Mixed-species biofilms containing integrated fungal components frequently demonstrate reduced susceptibility to antimicrobials and host-derived defenses (203). The fungal matrix reduces antimicrobial penetration, while interspecies stress responses upregulate efflux pumps and phenotypic tolerance mechanisms (44). Clinically, this cooperative resistance contributes to suboptimal outcomes following mechanical therapy and adjunctive antimicrobial treatments, highlighting the need to reconsider therapeutic strategies when fungal enrichment is present (108). Patients with elevated oral *Candida* loads are more likely to exhibit severe periodontal destruction and reduced responsiveness to standard therapy, as these mixed-species biofilms display recolonization dynamics that impair long-term disease control following mechanical therapy alone (101, 201).

### 5.2 Modulating interkingdom communication

A promising avenue for therapeutic innovation lies in modulating the sophisticated communication systems that underpin interkingdom interactions within oral biofilms (202). Quorum quenching, or quorum-sensing inhibition, represents a potential antimicrobial tool designed to attenuate the pathogenicity of oral biofilms by disrupting bacterial signaling networks (204). Quorum sensing constitutes a process by which bacteria chemically communicate through the synthesis and export of signaling molecules called autoinducers (205). Upon reaching a specific threshold concentration, these autoinducer molecules facilitate the ability of biofilm bacteria to perceive population density and coordinate activities, thereby promoting collective behavioral changes that enhance virulence in *in vivo* experiments (206). Quorum-quenching strategies aim to disrupt the communication networks responsible for virulence rather than directly killing the microorganisms. Research indicates that furanone compounds and D-galactose show promise as quorum-quenching agents against key periodontal pathogens, such as *Fusobacterium nucleatum*, *Porphyromonas gingivalis*, and *Tannerella forsythia*, and are associated with reductions in biofilm biomass and alveolar bone loss (206, 207).

Given the significant role of fungi, particularly *Candida albicans*, in enhancing biofilm virulence, creating anaerobic niches, and promoting bacterial invasion, targeted anti-fungal strategies represent an important therapeutic consideration (208). While comprehensive anti-fungal therapies specifically for periodontitis are still emerging, the application of combination therapies for interkingdom infections is being increasingly investigated (101). For instance, certain natural compounds, such as berberine, exhibit broad antimicrobial activity against various bacteria, fungi, and parasites—including specific oral pathogens—while also demonstrating potent anti-inflammatory effects (209).

### 5.3 Targeted microbial interventions

Beyond modulating interkingdom communication, targeted interventions aimed at restoring microbial balance or specifically eliminating pathogens offer sophisticated therapeutic adjuncts to traditional mechanical debridement. The administration of live microorganisms, known as probiotics, is being explored for its potential to support host health by promoting homeostasis within the oral microbiome (209). Probiotics modulate the microenvironment through several mechanisms: they compete with pathogens for resources and spatial niches, thereby inhibiting pathogen colonization and growth; they produce antimicrobial compounds, such as hydrogen peroxide and bacteriocins; and they modulate host immune responses to promote anti-inflammatory effects, thereby mitigating tissue damage and gingival inflammation (210). Investigations into the clinical efficacy of probiotics in periodontitis primarily focus on the capacity of these introduced species to regulate biofilm formation and mitigate dysbiosis (209).

An evaluation of a probiotic delivery system for the prevention of oral candidiasis demonstrated that *Lactobacillus paracasei* 28.4, when incorporated into gellan gum, exhibits significant antifungal and antibiofilm activity against *Candida albicans*. Through a series of *in vitro* assays, the authors showed that probiotic–gellan gum formulations maintained bacterial viability, facilitated sustained bacterial release, and significantly inhibited *C. albicans* growth, biofilm formation, and hyphal development in a dose-dependent manner. Structural analyses using scanning electron microscopy indicated disrupted fungal biofilm architecture with reduced hyphal elements, while functional assays confirmed decreased fungal cell counts and total biomass. Furthermore, *in vivo* murine studies demonstrated that oral administration of the probiotic formulation reduced the fungal burden, mitigated the development of candidiasis lesions, and attenuated mucosal inflammation compared with untreated controls. Collectively, these findings illustrate a clinically relevant cross-kingdom antagonistic interaction, in which probiotic bacteria attenuate fungal virulence within the oral niche, supporting the concept that targeted, biomaterial-assisted probiotic delivery represents a promising microbiome-based strategy to manage oral candidiasis while preserving oral microbial ecosystem balance (211).

Phage therapy, which utilizes bacteriophages to specifically target and treat bacterial infections, is experiencing renewed interest, particularly in the context of multidrug-resistant bacteria (212). Bacteriophages offer a highly specific approach, targeting particular bacterial species or strains without significantly impacting beneficial microbiota, thereby minimizing dysbiosis and reducing the collateral damage often associated with broad-spectrum antibiotics (213). Phages possess the capacity to penetrate biofilms, replicate within bacterial cells, and disrupt the biofilm matrix, positioning these viruses as effective agents against biofilm-associated infections (214). Furthermore, phage therapy can be combined with antibiotics to achieve synergistic effects, facilitating the circumvention of bacterial resistance and reducing the likelihood of future resistance development (215). In the context of periodontitis, the objective of phage therapy is to dismantle the dysbiotic bacterial community, thereby resolving inflammation and facilitating the reestablishment of a mutualistic subgingival ecosystem. Ultimately, these targeted microbial interventions—including both probiotics and phage therapy—prioritize the restoration of eubiosis over broad-spectrum sterilization, aligning with the contemporary understanding of periodontitis as a dysbiotic disease (216).

### 5.4 Beyond mechanical therapy: adjunctive and personalized approaches

While non-surgical mechanical therapy remains the foundational strategy for periodontitis, this approach is frequently insufficient, particularly in cases involving extensive periodontal pockets or significant architectural bone defects that require surgical intervention (217). Consequently, a comprehensive therapeutic paradigm extends beyond traditional debridement to incorporate a range of adjunctive and personalized strategies designed to mitigate the persistent interkingdom microbial challenge. For example, adjunctive therapies, such as ozonized water and oil, are utilized for their significant antimicrobial and anti-inflammatory properties, facilitating the reduction of the microbial burden and promoting tissue healing. These adjunctive modalities are designed to enhance the clinical outcomes of mechanical therapy by targeting the dysbiotic microenvironment that frequently persists following standard scaling and root planing (218).

Crucially, the future of periodontal management lies in personalized treatment strategies informed by advanced microbial analyses. Modern methods, particularly multi-omics approaches such as metagenomics, metatranscriptomics, and metabolomics, facilitate the identification of specific microbial profiles and functional activities associated with various phases of the disease (219). These technologies have significantly advanced the field by enabling the detection, functional inference, and systems-level analysis of interkingdom crosstalk within the subgingival microenvironment, although challenges related to data integration and clinical standardization remain. This analytical capability facilitates the development of individualized therapeutic regimens (219). Furthermore, biomarker-based diagnostic approaches—utilizing host-derived pro-inflammatory cytokines such as interleukin-1β (IL-1β) and tumor necrosis factor-α (TNF-α), alongside microbial DNA detected in saliva, blood, and gingival crevicular fluid—are being explored to monitor disease progression and treatment response (219). Because interkingdom dysbiosis within the subgingival microbiome is recognized as a key driver in the initiation and progression of periodontitis, the development of clinical tools capable of tracking these ecological shifts in real-time is essential (18).

Additionally, artificial intelligence (AI) is increasingly being utilized in periodontology to enhance diagnostic accuracy and clinical efficiency (179). AI, particularly through convolutional neural networks, has been applied to analyze visual imaging data for periodontitis diagnosis, simultaneously assess multiple biomarkers, identify complex disease patterns, and provide real-time diagnostic recommendations (220). This technological integration signifies a shift toward precision medicine in periodontology; rather than relying on a uniform clinical approach, future treatments will be tailored to the specific interkingdom dysbiosis and immune profile of each individual patient, facilitating more effective and targeted interventions (218). **Table 3** summarizes the most relevant strategies therapeutically targeting interkingdom interactions.

**Table 3:** Emerging therapeutic strategies targeting interkingdom interactions.

Therapeutic strategy	Mechanism of action	Interkingdom relevance	Current status/future potential	Examples
Quorum Quenching (QQ)	Inhibits bacterial communication (quorum sensing)	Targets bacterial-fungal/bacterial-bacterial communication within polymicrobial biofilms	Promising *in vitro* and murine model studies; clinical validation needed	Furanones, D-galactose
Anti-fungal Therapies	Disrupts fungal-bacterial synergy, reduces fungal burden	Addresses the role of fungi (e.g., *C. albicans*) in enhancing bacterial virulence and biofilm formation	General concept of combination therapy; specific periodontitis applications emerging	Berberine, combination antimicrobials
Probiotics	Restores microbial balance, produces antimicrobials, modulates immune response	Introduces beneficial bacteria/yeasts to outcompete pathogens and re-establish eubiosis	Investigated for beneficial effects; further studies needed for definitive conclusions	*Lactobacillus* spp.
Phage Therapy	Specific bacterial lysis, biofilm disruption, reduces antibiotic resistance	Targets specific bacterial hosts within the polymicrobial community	Regaining momentum, promising against MDR bacteria; requires more isolation/characterization	Specific bacteriophages targeting periodontopathogens
Adjunctive Ozonized Materials	Significant antimicrobial and anti-inflammatory properties	Reduces overall microbial burden (bacteria, viruses, fungi) and promotes tissue healing	Clinical research shows significant improvements in periodontal parameters	Ozonized water, ozonated oil
Personalized Treatment	Tailored to individual microbiome and host response profiles	Utilizes advanced microbial analyses and biomarkers for precise intervention	Emerging with high-throughput sequencing and AI; marks precision periodontal medicine	AI-driven diagnostics, biomarker-guided therapy

QQ, Quorum Quenching; MDR, Multidrug-Resistant; AI, Artificial Intelligence.

## 6 Conclusion and future perspectives

The transition from a bacteria-centric model to an interkingdom paradigm necessitates a fundamental re-evaluation of translational periodontology (21, 202). Because the systemic implications of oral dysbiosis contribute directly to various non-communicable diseases, future therapeutic paradigms must extend beyond conventional mechanical debridement to actively modulate multikingdom communication systems (160, 203). Developing targeted microbial interventions that address the entire periodontal ecosystem holds significant potential to mitigate the intricate interrelationships between periodontitis and systemic comorbidities (221).

Despite these promising translational implications, critical research gaps define the field’s future trajectory. A fundamental unresolved question is the precise molecular mechanisms governing viral and archaeal interactions within the subgingival niche *in vivo*. Addressing this requires overcoming current methodological limitations through the expanded application of longitudinal multi-omics studies, specifically integrating metagenomics, metatranscriptomics, and metabolomics. Such longitudinal tracking is essential to capture dynamic shifts in interkingdom communities over time, particularly in response to environmental factors, systemic health changes, and targeted therapies.

Furthermore, while novel therapeutic strategies such as phage therapy and quorum quenching demonstrate theoretical promise, these approaches require large-scale, well-designed clinical studies to validate their efficacy and assess their long-term ecological impacts on the subgingival microenvironment (204). Finally, realizing the full potential of precision medicine in periodontology will require the development and widespread clinical implementation of rapid, multiplex biomarker detection platforms and AI-driven diagnostics (218). Addressing these priorities will facilitate the development of holistic, preventive strategies that successfully integrate the mycobiome, virome, and archaeome into personalized periodontal care (108).
