# Supplementary material for: Host-pathogen interactions in periodontitis: an integrative interkingdom perspective
Source: Front Immunol. 2026 Apr 2;17:1797726. doi: 10.3389/fimmu.2026.1797726 (PMC13083134; doi:10.3389/fimmu.2026.1797726)
Supplement: Supplementary file 1 [file DataSheet1.pdf]

PRISMA-S STATEMENT

This literature search and reporting process followed the PRISMA-S extension to ensure transparency and reproducibility. A structured search strategy was developed to identify evidence addressing interkingdom host–pathogen interactions, polymicrobial dysbiosis, and the systemic implications of periodontitis.

Electronic database searches were conducted using predefined combinations of controlled vocabulary and free-text terms related to *periodontitis*, *oral microbiome*, *polymicrobial dysbiosis*, *interkingdom interactions*, *host–pathogen interactions*, *biofilms*, *mycobiome*, *virome*, *archaeome*, and *systemic disease associations*. Boolean operators, truncation, and field restrictions were applied where appropriate to optimize sensitivity and specificity. Search filters were used to prioritize peer-reviewed articles and studies involving human, animal, and translational experimental models.

The core search strategy used for database queries was structured as follows: ("periodontitis"[MeSH Terms] OR "periodontal disease*" OR periodontitis) AND ("oral microbiome" OR "oral microbiota" OR "oral microbiology") AND ("host-pathogen interaction*" OR "host microbe interaction*" OR dysbiosis OR "polymicrobial synergy") AND (interkingdom OR "cross-kingdom" OR "microbial interaction*" OR biofilm*) OR (mycobiome OR fungi OR *Candida*) OR (virome OR virus* OR bacteriophage*) OR (archaea OR archaeome).

To ensure conceptual completeness, supplementary search methods included backward and forward citation tracking and manual screening of reference lists from seminal and recent publications.

Eligibility criteria were defined a priori. Studies were included if they:
(1) were peer-reviewed original investigations or evidence syntheses;
(2) examined multikingdom microbial communities (bacteria, fungi, viruses, and archaea) in periodontal health or disease;
(3) addressed ecological dysbiosis, interspecies signaling, biofilm dynamics, or host–microbiome interactions; and
(4) provided mechanistic, clinical, or translational insight relevant to periodontal pathogenesis and systemic implications.

Studies were excluded if they focused solely on single-pathogen models without an ecological context or lacked sufficient methodological rigor.

All records were managed and screened using Rayyan systematic review software. Title and abstract screening were conducted before full-text eligibility assessment of potentially relevant studies.

To enhance methodological transparency, a cross-verification process was conducted between the final manuscript reference list and the Rayyan screening database. This process confirmed alignment between screened and cited studies, with minor discrepancies attributable to recently published literature and methodological references incorporated during manuscript development.

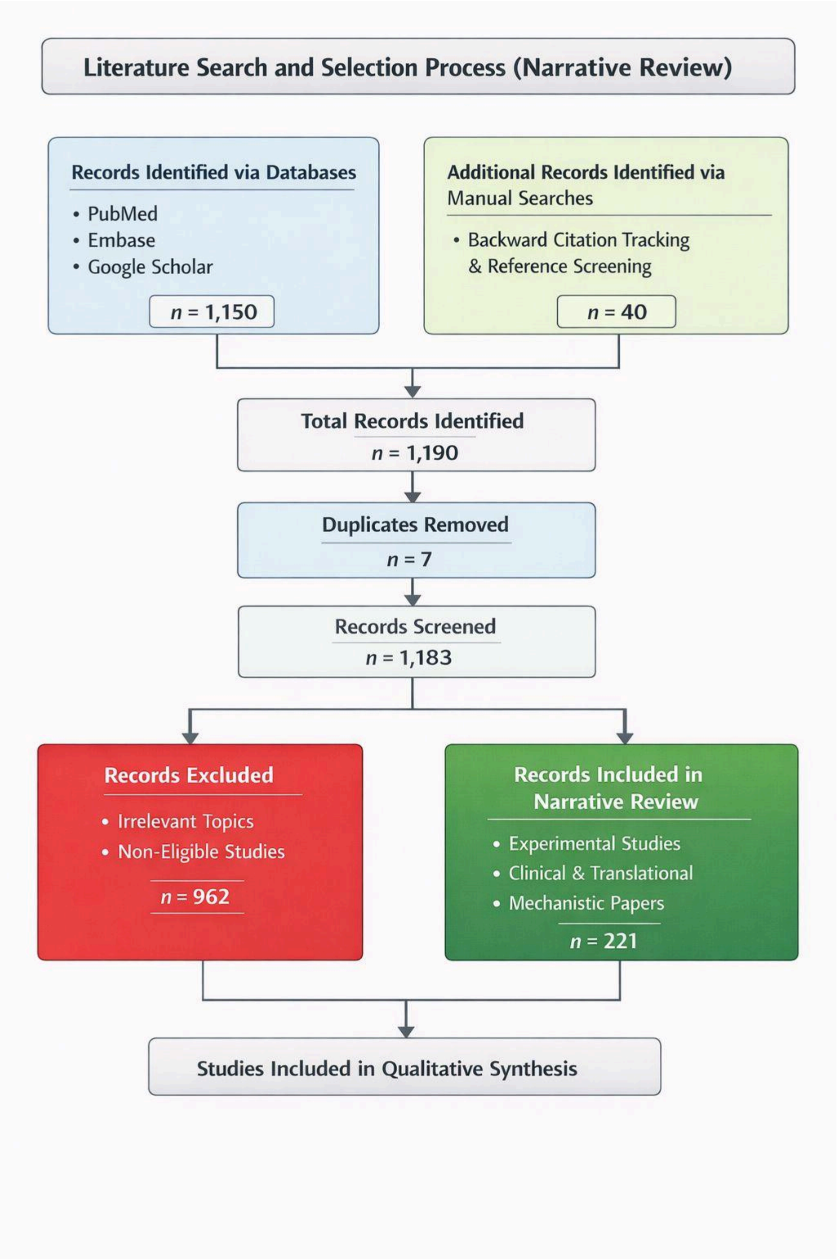

Figure S1. PRISMA-style flow diagram adapted for a narrative review. Flowchart illustrating the literature search and study selection process.
